# Pyramiding of High Grain Weight With Stripe Rust and Leaf Rust Resistance in Elite Indian Wheat Cultivar Using a Combination of Marker Assisted and Phenotypic Selection

**DOI:** 10.3389/fgene.2020.593426

**Published:** 2020-12-22

**Authors:** Satinder Kaur, Jaspreet Kaur, G. S. Mavi, Guriqbal Singh Dhillon, Achla Sharma, Rohtas Singh, Urmila Devi, Parveen Chhuneja

**Affiliations:** ^1^School of Agricultural Biotechnology, Punjab Agricultural University, Ludhiana, India; ^2^Department of Plant Breeding and Genetics, Punjab Agricultural University, Ludhiana, India

**Keywords:** wheat, PBW550, PBW343, pyramiding, grain weight, rust resistance

## Abstract

Wheat (*Triticum aestivum* L.) is an important cereal crop globally as well as in India and yield improvement programs encounter a strong impediment from ever-evolving rust pathogens. Hence, durable rust resistance is always a priority trait for wheat breeders globally. Grain weight, represented as thousand grain weight (TGW), is the most important yield-contributing trait in wheat. In the present study high TGW has been transferred into two elite Indian wheat cultivars PBW343 and PBW550 from a high TGW genotype, Rye selection 111, selected from local germplasm. In the background of PBW343 and PBW550, an increase in TGW upto 27.34 and 18% was observed, respectively (with respect to recipient parents), through conventional backcross breeding with phenotypic selections in 3 years replicated RBD trials. Resistance to leaf rust and stripe rust has been incorporated in the high TGW version of PBW550 through marker assisted pyramiding of stripe rust resistance gene *Yr15* using marker *Xuhw302*, and a pair of linked leaf rust and stripe rust resistance genes *Lr57-Yr40* using marker *Ta5DS-2754099_kasp23*. Improved versions of PBW550 with increased TGW ranging from 45.0 to 46.2 g (up to a 9% increase) and stacked genes for stripe and leaf rust resistance have been developed. This study serves as proof of utilizing conventional breeding and phenotypic selection combined with modern marker assisted selection in improvement of important wheat cultivars as a symbiont of conventional and moderan techniques.

## Introduction

Wheat (*Triticum aestivum* L.) is an important cereal crop in India, ranking second after rice for the area (29.31 million hectares) and production (103.6 million metrics) with the state of Punjab sharing 18% of production. With global per capita consumption of 67.4 kg/year, wheat is the most widely consumed food grain (Djanaguiraman et al., 2019). In India only, the population is projected to cross the 1.70 billion mark by 2050 with a domestic demand of wheat exceeding 140 million tons (Nagarajan, 2005)^1^. A consistent increase in the wheat yields is a primary goal for food security of the growing population (Singh et al., 2007; Ye and Smith, 2008). Crop yield is a complex quantitative trait determined by different parameters of tiller number, grain number, grain weight, etc. Grain or kernel weight (1,000 grain weight in g, TGW), consisting of grain length, width, and area has high heritability (> 0.68) (Giura and Saulescu, 1996; Kuchel et al., 2007b) which not only translates into higher yields but also has a favorable effect over flour yield (Gegas et al., 2010). Moreover, uniform and larger sized grains are visually appealing and fetch higher market prices.

As rusts are major diseases in India, keeping pace of increasing yields with rust resistance is one of the most challenging tasks. The three species of rust viz., stripe rust caused by *Puccinia striiformis*, leaf rust caused by *Puccinia triticina*, and stem rust caused by *Puccinia graminis* are severely affecting wheat yield. An estimated loss of 200 million rupees occur every year due to rusts (Mehta, 1950). Leaf rust is prevalent in all the wheat growing zones of India and its widespread occurrence was observed during periods of 1971–1973, 1993–1994 (Joshi et al., 1975; Nayar et al., 1997). The 70% of the total area under wheat cultivation in cooler parts Northern India is under constant threat of ever evolving epidemic stripe rust pathogenic races. It occurred almost every year from 1967 to 1974, with high incidence recorded in 2001 and 2011 (Nayar et al., 1997; Prashar et al., 2007; Pannu et al., 2010; Tomar et al., 2014; Pal et al., 2015). An approximate loss of rupees 236 crore have been recorded in Punjab state during the epidemic year of 2009–2010 (Jindal et al., 2012). Stem rust on other hand is important central and peninsular India since its first epidemic reported in 1786AD (Nagarajan and Joshi, 1975).

Despite the devastating nature and continuous occurrence of rusts, wheat production is growing linearly due to the continuous addition of new rust resistance genes in the wheat gene pool. Use of resistant wheat cultivars is not only effective and economical but also environment friendly (Peng et al., 2003; Singh et al., 2020). Due to pathogen evolution, combination of resistance genes is being pursued by the crop breeders world over for increasing durability of resistance. Resistance gene pyramiding has been found to increase the life of each gene though the synergistic effect of pyramided genes (Klymiuk et al., 2018; Mundt, 2018). One of the greatest successes in the history of resistance breeding is with the pyramiding of resistance genes in controlling stem rust (Singh et al., 2015). The Ug99 race group of pathotypes of stem rust defeated rust resistance genes *Sr31* and *Sr38* but their differential pyramiding combinations of different resistant genes *Sr22*, *Sr25*, *Sr26*, *Sr33*, *Sr35*, *Sr45*, and *Sr50* were found to be effective. For leaf rust, pyramiding of different genes in combination with leaf rust resistance genes provided long lasting resistance (Kolmer, 1996; Bhawar et al., 2011; Aboukhaddour et al., 2020; Babu et al., 2020). Resistance to stripe rust has also been significantly improved by pyramiding of different resistance genes (Zheng et al., 2017; Randhawa et al., 2019).

The present study reports the introgression of high thousand grain weight (TGW) to wheat varieties PBW343 and PBW550 from a local selection named “Rye Selection 111.” Pyramiding of three rust resistance genes viz *Yr15* and linked *Lr57-Yr40* into improved TGW version of PBW550 was done using “transfer first and assemble later” strategy (Ishii and Yonezawa, 2007a,b). Wheat cultivar PBW343, released in India in 1995, was the most widely grown cultivar in the country and soon was recognized as the new miracle genotype with wider adaptability and yield potential (Gupta et al., 2018). PBW550 was released in 2008 and farmers’ receptivity to this short duration variety with bold grains has accelerated its spread in the first few years of its release (Gupta et al., 2018). However, both of these hexaploid elite cultivars succumbed to emerging races of YR thus it became imperative to restructure the genetic makeup of these cultivars for improved yield and rust resistance.

## Materials and Methods

### Plant Material

The plant material used in the present study included two cultivated hexaploid bread wheat varieties named PBW343 (ND/VG9144//KAL/BB/3/YACO’S’/4/VEE#5 “S,” notification number 1(E) dated 01.01.1996, Indian Council of Agricultural Research, Government of India) and PBW550 (WH 594/RAJ 3856//W 485 notification number 72(E) dated 10.01.2008, Indian Council of Agricultural Research, Government of India), two near isogenic lines (NILs) developed at Punjab Agricultural University (PAU) in PBW550 background viz PBW550 + *Yr15* and PBW550 + *Lr57-Yr40*, a high thousand grain weight selection from local germplasm named Rye selection 111 (RyeSel 111) and the progenies from crosses of RyeSel 111 × PBW343, RyeSel 111 × PBW550, HGW343 × PBW550, HGW550 × PBW550 + *Yr15* and HGW550 + *Yr15* × PBW550 + *Lr57-Yr40*. (The “PBW” stands for Punjab Bread Wheat followed by numeric value of varietial release number, HGW = high grain weight).

### Introgression of High Grain Weight in PBW343 and PBW550

**FIGURE 1:**
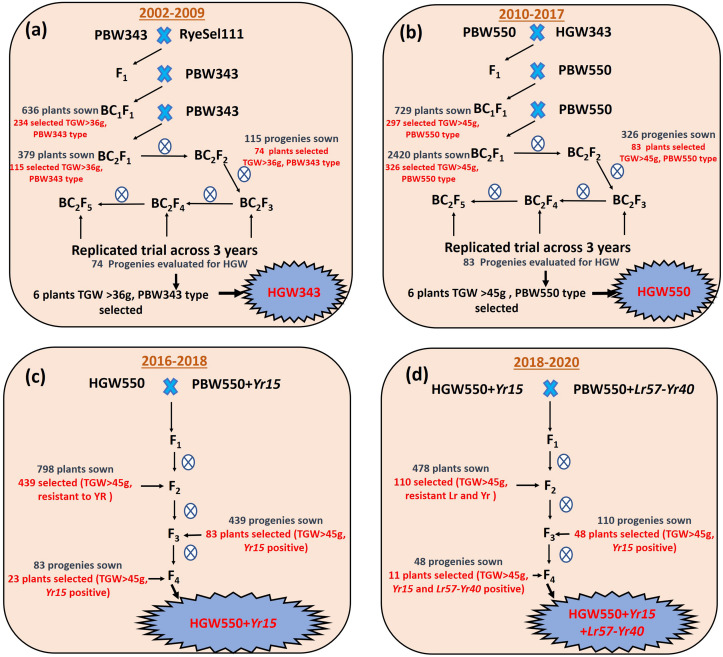
Schematic view of the transfer of high thousand grain weight to **(a)** wheat variety PBW343 to obtain HGW343. **(b)** Wheat variety PBW550 to obtain HGW550. **(c)** Combining HGW550 with stripe rust resistance gene *Yr15*. **(d)** Combining HGW550 + *Yr15* with linked leaf rust and stripe rust resistance gene *Lr57-Yr40.*

For transfer of high TGW to wheat variety PBW343, cross was made with RyeSel 111 as male parent and the phenotypic selection was done in subsequent backcross generations (Figure 1a). The BC_2_F_5_ progenies of cross PBW343-RyeSel 111 with high TGW will be referred to as HGW343 hereon. Selected HGW343 plants were used as donor to transfer high TGW to wheat variety PBW550 and BC_2_F_5_ progenies with high TGW (Figure 1b) thus obtained will be termed as HGW550 hereon. All the crosses and selections were done at PAU, Ludhiana, India.

### Phenotyping and Experimental Design

In both the above crosses, traits were recorded on single plants in BC_2_-F_1_/F_2_ generations while in BC_2_-F_3_/F_4_/F_5_ progenies, traits were recorded on 10 single plants of each progeny as well as bulk of progeny. Single plants from uniform and promising progenies were selected and carried forward. Evaluation of each of the BC_2_-F_3_/F_4_/F_5_ progenies along with parental lines were done in three replications (1.5 m paired rows with the plant to plant distance of 10 cm and row to row distance of 20 cm) in randomized block design. Wherever single plant selections were done, the weight of 300–500 grains (as available per plant) was recorded and converted into TGW, while the weight of thousand grains was recorded for progeny bulks. Selections were done for plant/progenies having TGW higher than recurrent parent. Similarly other phenotypic traits of plant height, tiller number, spikelets per spike, spike length, and grains per spike were also recorded and plants/progenies having either similar value to or higher than recurrent parent were selected (data not given).

For each progeny the mean values were calculated for recorded traits in each replication and adjusted means of replications were finalized for each year. Three years data was again used to calculate adjusted means across the years for final selections.

### Combining High Grain Weight and Rust Resistance in PBW550

For pyramiding HGW with stripe rust resistance gene *Yr15*, first the selected HGW550 progenies obtained from the above cross were crossed with NIL PBW550 + *Yr15*. The HGW550 + *Yr15* thus obtained was again crossed with another NIL, PBW550 + *Lr57-Yr40*, to develop the HGW550 + *Yr15* + *Lr57*-*Yr40* line. Selections were done for high TGW and rust resistance as for single plant in F_1_/F_2_ generations, while in F_3_/F_4_/F_5_ progenies 10 single plants along with progeny bulks recorded to select single plants from best performing progenies. Since both the parental lines were from the same background, no backcrossing was done.

### Marker Assisted Selection for *Yr15* and *Lr57-Yr40* Genes

Selection for rust resistance was done in a combined phenotypic–genotypic manner. For marker assisted selection, DNA from parental lines and segregating progenies was extracted using CTAB method (Saghai-Maroof et al., 1984) with the small modification of reducing incubation time to 30 min at 65°C with CTAB buffer and to 20 min at room temperature for solvent extraction using chloroform: isoamyl alcohol (24:1). *Yr15* positive plants were selected by amplifying gene specific marker *Xuhw302* (Klymiuk et al., 2018). For the selection of *Lr57*-*Yr40* gene, linked Kasp marker *Ta5DS-2754099_kasp23* (Bansal et al., 2020) and caps marker *Lr57-Yr40_caps16* (Toor et al., 2016) were used. The PCR reaction for gel based marker *Xuhw302* and *Lr57-Yr40_caps16* was done in 10 μl reaction volume (60 ng DNA, 5 μl of 2× EmeraldAmp^®^ GT PCR Master Mix, 0.75 μl of 5 μM each primer) in 384 well microtiter plate in an Applied Biosciences 384 thermal cyclers. The PCR products were resolved using 2.5% agarose gel electrophoresis and visualized and photographed using gel documentation system. The scoring was done identifying the presence of gene specific amplicon as per the positive control. The Kasp marker *Ta5DS-2754099_kasp23* was amplified in 4 μl reaction (20 ng DNA, 1.944 μl of 2X KASP V3.0 master mix from LGC, Biosearch Technologies, 0.056 μl of primer mix in ratio of 12:12:30:46 Allele specific Primer I:Allele specific II primer: Common primer: Water) in a 384 well microtiter plate. The amplicons were identified by measuring allele specific flourescence in a high throughput TECAN infinite F200 PRO plate reader. Kluster Caller software was used to view the allele specific calls in an *x–y* plot to identify the positive and negative allels against the positive control.

### Screening Against Stripe Rust and Leaf Rust

Screening for stripe rust and leaf rust was also done in the field along with marker selection to validate the effectiveness of gene pyramiding. F_3_ and F_4_ progenies of HGW550 X PBW550 + *Yr15* were screened against stripe rust at the adult plant stage in the field during season 2016–2017 and 2017–2018. Similarly, F_3_ and F_4_ progenies of HGW550 + *Yr15* X PBW550 + *Lr57*-*Yr40* were screened both against stripe rust and leaf rust at the adult plant stage in the field during season 2018–2019 and 2019–2020. For screening, artificial epiphytotic conditions for rust were created by spraying the urediniospores mixed and diluted in water, containing Tween-20, of *Pst* pathotypes (100S119, 78S84), and *Pt* pathotypes (77-1, 77-2, 77-5, 104-2) and a mixture of leaf rust and stripe rust inoculum collected from farmers’ fields. For the uniform spread of disease, highly susceptible cultivar WL711 was planted as spreader rows all around the field and after every 20 rows. Rust data was recorded when WL711 showed complete susceptibility. Rust was recorded using Cobbs scale, as illustrated in McIntosh et al. (1995) where types of spores were recorded as zero (immune); TR (traces of severity); MR (moderately resistant), MS (moderately susceptible); and S (susceptible), and numeric numbers associated with these scores signified the percentage of leaf area covered by rust. The TR score is given to a plant when there are no lesions, MR when there are no visual postules of spores on the leaf but apoptotic lesions are visible, MS when there are minute visual postules of spores on the leaf with apoptotic lesions while *S* score is given to a plant when advanced postules of spores are visible to the leaf.

## Results

### Development and Selection of HGW343

Different number of plants phenotypically similar to PBW343 with TGW > 36g were selected from the cross of PBW343-Rye Sel 111 across different generations. In the crop season 2003–2004, 636 BC_1_F_1_ plants were sown and BC_2_ progenies of only 234 BC_1_F_1_ plants (Figure 1a) were carried forward. In BC_2_F_1_, 115 plants were selected and planted as single plant-to-row BC_2_F_2_ progenies. During the years 2005–2006, from 943 well-established BC_2_F_2_ single plants, only 74 superior plants were selected.

**FIGURE 2:**
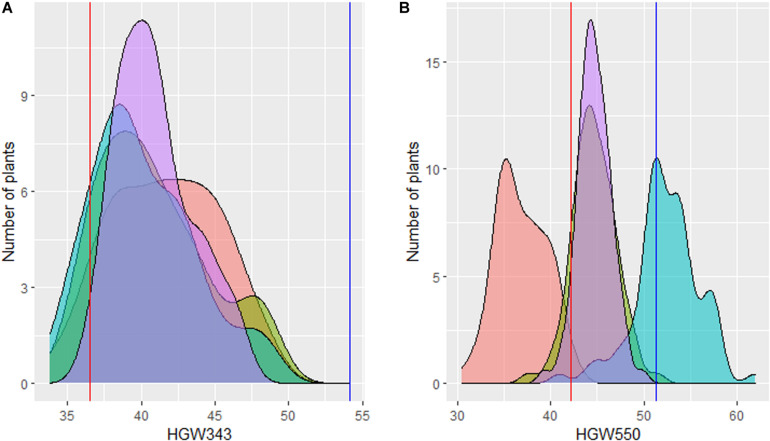
Graphical representation of distribution of thousand grain weight of derived BC_2_F_3__–__5_. **(A)** HGW343 progenies and **(B)** HGW550 progenies with the BLUPs in each environment (purple). The BLUPs of Rye Selection111 and recurrent parents (PBW343 and PBW550) are given as vertical red lines and blue lines, respectively.

**FIGURE 3:**
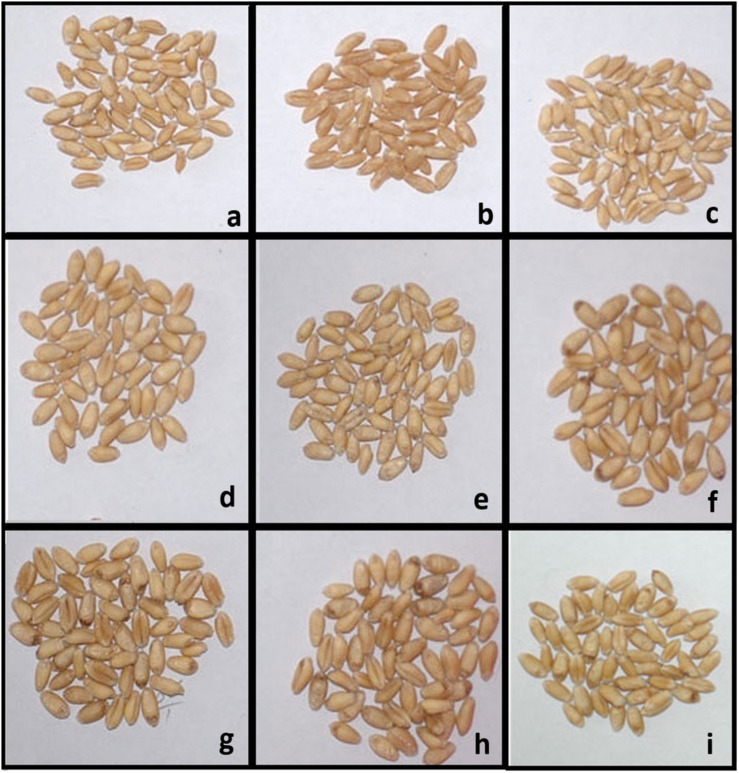
Representative grains from the field grown BC_2_F_5_ progenies along with parental genotypes. **(a)** PBW343. **(b)** Rye selection 111. **(c–i)** BC_2_F_5_ progenies obtained from cross of PBW343/Rye selection 111.

For three consecutive seasons (2006–2009), 74 BC_2_F_3__–__5_ progenies, along with RyeSel 111 and PBW343, were evaluated in replicated trials. Adjusted means for 3 years showed that TGW ranged from 36.66 to 46.81 g (0.30–28.10% increase), where TGW for PBW343 was 36.54 g and for RyeSel 111 was 54.17 g (Table 1 and Figure 2). Six BC_2_F_5_ plants of progenies with significantly high TGW, namely HGW343-3 (TGW-45.36 g, 24.13% increment), HGW343-6 (TGW-45.54 g, 24.63% increment), HGW343-8 (TGW-46.08 g, 26.11% increment), HGW343-60 (TGW-46.48 g, 27.20% increment), HGW343-61 (TGW-46.71 g, 27.83% increment), and HGW343-11 (TGW-46.81 g, 28.10% increment) were selected (Figure 3). The popularity of PBW343 in the late 1990s and early 2000s led to its widespread sowing which facilitated the selection of virulence for a super aggressive stripe rust race *78S84* (Prashar et al., 2007), breaking down resistance of its predominant gene *Yr27*, crumbling the wheat production in India. For restructuring the resistant version of PBW343, the high TGW progenies in PBW343 background were selected and disease resistance gene pyramiding carried out under a separate breeding program (not included in the present manuscript).

**TABLE 1:** Statistical analysis of HGW343 and HGW550 progenies across F_3_, F_4_, and F_5_ generations planted in RBD design.

	HGW343	HGW550
RP	36.54	42.23
RyeSel111	54.17	51.36
Range	36.66–48.36	39.29–49.83
Heritability	0.85	0.76
Genotype variance	11.52	4.88
Residual variance	5.89	4.5
Grand mean	41.11	44.74
LSD	3.62	2.99
CV	5.9	4.74
*n* replicates	3	3
*n* years	3	3

**RP, recurrent parent; LSD, least significant difference; CV, coefficient of variation.**

### Development and Selection of HGW550

**FIGURE 4:**
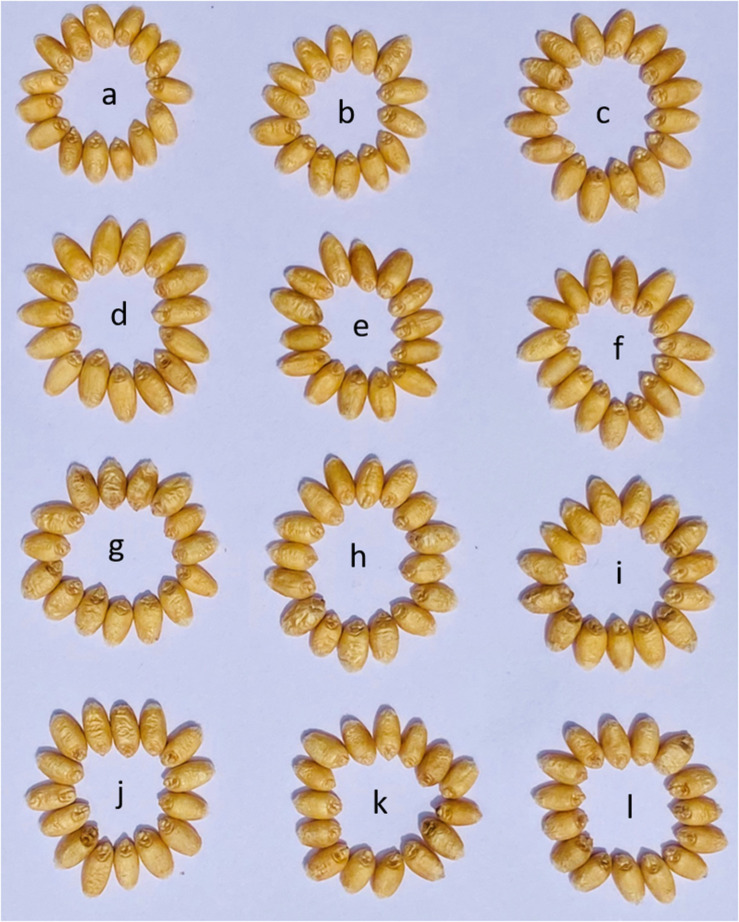
Representative grains from the field grown pyramided lines along with parental genotypes. **(a)** PBW550. **(b)** PBW550 + *Yr15*. **(c)** PBW550 + *Yr15* + *Lr57-Yr40.* **(d–f)** BC_2_F_5_ progenies obtained from cross of HGW343 × PBW550. **(g–i)** F_4_ progenies obtained from cross of HGW550 × *Yr15*; **(j–l)** F_4_ progenies obtained from cross of HGW550 + *Yr15* X *Lr57-Yr40.*

Four HGW343 plants named HGW343-8, HGW343-60, HGW343-61, and HGW343-11 were crossed and backcrossed with PBW550 (Figure 1b). Phenotypic selections for high TGW were done in BC_1_F_1_, BC_2_F_1_, and BC_2_F_2_. From 729 BC_1_F_1_ plants, backcrosses from 297 single plants were selected to obtain 2,420 BC_2_F_1_ plants. A total of 83 BC_2_F_2_ plants were selected and BC_2_F_3_, BC_2_F_4_, and BC_2_F_5_ progenies were evaluated across 3 years (2014–2017) in replicated trials, each having three replications (Table 1 and Figure 2). Overall adjusted means for 3 years showed that TGW for the progenies ranged from 39.29 to 49.83 g where TGW for PBW550 was 42.23 g and for RyeSel 111 was 51.36. Six plants from progenies with TGW higher than 47.0 g, namely HGW550-8 (TGW-49.83 g, 17.99% increment), HGW550-6 (TGW-48.1 g, 13.90% increment), HGW550-63 (TGW-47.93 g, 13.50% increment), HGW550-3 (TGW-47.64 g, 12.81% increment), HGW550-7 (TGW-47.54 g, 12.57% increment), and HGW550-21 (TGW-47.5 g, 12.48% increment) were selected (Figure 4) for introducing rust resistance.

### Marker Assisted Pyramiding of HGW550 and *Yr15*

Three selected HGW550 plants; HGW550-8, HGW550-6, and HGW550-63 were crossed with NIL PBW550 + *Yr*15 (Figure 1c). F_1_s thus obtained were selfed to develop 798 F_2_ plants and 439 stripe rust resistant plants with TGW of more than 42 g were selected. In F_3_, plants having a TGW equal to or more than 45.0 g were selected from promising uniform stripe rust resistant progenies. The selected plants were screened with molecular marker *Xuhw302*, and 83 plants, homozygous for *Yr15* were selected (Supplementary Figure S1A). These 83 plants were further sown in plant to progeny rows in F_4_ and 23 single F_4_ plants with TGW ranging between 45.0 and 48.0 g and positive for *Yr15* were selected (Figure 4). These plants were also completely resistant to stripe rust in the field.

### Marker Assisted Pyramiding of HGW550 + *Yr15* and *Lr57*-*Yr40*

F_1_s generated by crossing four selected single F_4_ plants HGW550 + *Yr15*-34 (TGW-46.3), HGW550 + *Yr15*-29 (TGW-47.1), HGW550 + *Yr15*-3 (TGW-47.3), HGW550 + *Yr15*-34 (TGW-47.4) with PBW550 + *Lr57-Yr40* were selfed to generate 478 F_2_ plants, of which 110 individual F_2_ plants were selected with TGW > 45 g (Figure 1d). These 110 plants were sown as plants to progeny rows and 48 single F_3_ plants with the positive allele for *Yr15* and *Lr57-Yr40* markers were selected (Supplementary Figure S1B). These plants were also screened against stripe rust and leaf rust at the adult plant stage and found to be completely resistant to both diseases. These 48 plants were again evaluated in F_4_ generation in plant to row progenies and 11 F_4_ plants from superior progenies with TGW between 45.0 and 46.2 g and complete resistance to stripe rust and leaf rust have been selected (Figure 4).

## Discussion

The stacking of genes governing multiple traits enhances the value of breeding material besides improving its durability. Yield is a trait of foremost priority for the commercial success of a variety, but combining improved grain yield with disease resistance, and high grain quality is a need of the changing time and environment. The introgression and pyramiding of major genes/QTL for different traits through marker-assisted selection (MAS) has been reported in wheat (Gupta et al., 2010; Kumar et al., 2010; Tyagi et al., 2014; Gautam et al., 2020).

We used RyeSel 111 as the donor to transfer the high TGW trait to elite cultivar PBW343 and PBW550 through conventional phenotypic selection. Though QTLs for high grain weight have been reported in the RyeSel 111-Chinese Spring RIL population (Kumar et al., 2006), the reported linked markers were found to be non-polymorphic between the receipient and donor combinations used in the present study. Thus a conventional route is followed through phenotypic selection for high TGW. Mapping and transfer of QTLs for yield related traits often sound more logical theoretically, but these complex traits are controlled by many QTLs of large and small effects and pyramiding all these QTLs in one background through MAS to retrieve the donor effect is not always achieved. This is more often pronounced in cases where the minor QTLs additively contribute toward the trait along with major ones. Thus phenotypic selection especially for an easily quantified trait like grain weight, gives more realistic results. Wheat yield is controlled by traits of yield per area (includes grains per spike, grain weight, and spikes per area) and yield per spike (spikelet number per spike, grain number, and grain size) (Slafer et al., 2014). Several QTLs related to grain yield related traits have been mapped on different chromosomes but none have been cloned or effectively utilized for marker assisted selection. From different factors influencing wheat yield, grain weight was found to be one of the most stably inherited (Sidwell et al., 1976; Kuchel et al., 2007a,b; Aisawi et al., 2015) suggesting selection for heavier grains lead to effective yield improvement. Raising plant yield by conventional breeding methods (Reynolds et al., 2009) has remained very successful in the past, though new emerging technologies are creating a different niche. High TGW was introgressed effectively into two popular wheat cultivars cv. PBW343 and PBW550 through the phenotypic selection, and improved progenies with an increase of TGW by 27.34 and 18% were obtained, respectively. Initially, direct crosses of RyeSel 111 were done with PBW343 then using improved HGW343 progenies as the donor, HGW introgressed into PBW 550. Similarly improvement in grain yield by improving TGW has been reported by several studies (Giunta et al., 2007; Beche et al., 2014; Qin et al., 2015; Zhang et al., 2016; Gao et al., 2017).

PBW343 has been one of the most popular wheat varieties and was grown in about 25% of the 27 million hectares under the wheat cultivation in the country and contribute roughly 55% of the total wheat output in the country (Pavithra et al., 2017). Due to its wider adaptability to a range of environments, it has been a variety of choice for many improvement programs and was selected in this program for improving grain weight. After the PBW343 succumbed to *78S84* pathotype of stripe rust in 2004 and so did the whole breeding pipeline at PAU, it became mandatory to quickly mobilize some known stripe rust resistance genes to PBW343 and other advance germplasms to have a rust resistant variety for the farmers of the region. It was then that the development of rust resistant version of PBW343 became the main mandate of the wheat breeders, and reintroduction of its rust resistant version led to the shift of this work to a separate program.

PBW550, on the other hand, has been recognized as a good quality cultivar with bold grains and was spread to whole wheat growing regions of the country in the few years since its release in the year 2008. This variety was tested and also recommended for cultivation in Eastern and Central India for processing the commercial wheat flour under the PAU-ITC (Indian Tobacco Company) agreement. However, the evolution of the 78S84 pathogen was so targeted and led to the development of a very aggressive strain which not only rendered PBW343 susceptible to stripe rust but also slowly wrapped up PBW550 including all the newly released varieties during that time. As a result, PBW550 too became susceptible to yellow rust after 6–7 years of release despite having multi-pathogen resistance gene loci *Lr34/Yr18/Sr57*. The short-lived resistance makes it of utmost importance to introgress rust resistance in addition to yield improvement in these cultivars.

Though HGW has been introduced with backcross breeding only while the rust resistance was facilitated through MAS. Increased adoption of combining conventional breeding with the MAS approach (Gupta et al., 2010) in recent years has given multifold advantages, the major one being accelerated mobilization of the desirable gene(s) and their efficient stacking in elite backgrounds. Two different NILs carrying stripe rust resistance gene *Yr15* and linked leaf rust-stripe rust resistance gene *Lr57-Yr40* have been developed by PAU. NIL PBW550 + *Yr15* has also been released as a variety for cultivation in Punjab under timely sown irrigated conditions as *Unnat* PBW550. *Yr15* mapped near distal Nor-B1 on the short arm of chromosome 1B was first discovered in the 1980s in wild emmer wheat *T. turgidum var. dicoccoides*. Avocet + *Yr15* was initially used as a donor to transfer this gene in PBW550 background. As per the Global Rust Reference Centre^2^, *Yr15* has been providing a complete and broad spectrum resistance (Klymiuk et al., 2018). Linked *Lr57-Yr40* (on chromosome 5DS) has been transferred from an introgression line INGR15046, developed in the wheat wide hybridization program of PAU by transfer of leaf rust and stripe rust resistance from *Aegilopes geniculata* to wheat cultivar WL711 (Kuraparthy et al., 2007). This linked gene provides complete resistance to leaf rust and partial resistance (20MR) to stripe rust in field.

For pyramiding, the “transfer first and assemble later” approach (Ishii and Yonezawa, 2007a,b) already given above was followed where PBW550 + *Yr15* and PBW550 + *Lr57-Yr40*, NILs were used as donors of respective genes. Since both the parental genotypes were in the PBW550 background, backcrossing was not required. Similarly, HGW550 + *Yr15* and PBW550 + *Lr57-Yr40* were crossed, and selfing generations F_2_, F_3_, and F_4_ were evaluated for HGW and two resistances to leaf and stripe rust leading to the selection of 11 HGW550 + *Yr15* + *Lr57-Yr40* progenies with three rust resistance genes and high TGW. Gene pyramiding of disease resistance genes reported improving the durability of resistance, though the combined effect depends upon the nature of the individual genes and their synergism. Resistance breeding by marker led pyramid in the last decade is being used in wheat programs globally. Moreover, the combination of major and minor genes was also found to have more significant effects. Although *Yr15* has already succeeded in conferring stripe rust resistance for many years in different introgression lines around the world, marker assisted gene pyramiding of *Yr15*, and *Lr57-Yr40* genes provides durable resistance to stripe rust in combination with leaf rust resistance. Advanced breeding lines of PBW550 with three rust resistance genes and high TGW can lead to the development of new varieties and also serve as valuable germplasm for breeders to be used in the varietal development program to aid future breeding programs.

## Data Availability Statement

The original contributions presented in the study are included in the article/Supplementary Material, further inquiries can be directed to the corresponding author/s. The raw data for trials has been added as Supplementary Table S1.

## Author Contributions

SK conceived and designed the research, planned, and conducted the experiments, did rust screening, compiled the work, and produced the final draft of the manuscript. JK and RS conducted field experiments and did marker assisted selection. GM and AS conducted the field experiments. GD did statistical analysis and compilation of the manuscript. UD helped in field experiments. PC conceived and designed the research, and reviewed the manuscript. All authors contributed to the article and approved the submitted version.

## Conflict of Interest

The authors declare that the research was conducted in the absence of any commercial or financial relationships that could be construed as a potential conflict of interest.

## Footnotes

1 https://www.thehindubusinessline.com; https://www.eastasiaforum.org

2 https://agro.au.dk/forskning/internationale-platforme/wheatrust/

## Supplementary Material

Supplementary Figure 1 **(A)** PCR amplification profile of marker *Xuhw302* amplifying *Yr15* gene in F_3_ plants derived from cross HGW550 × PBW550 + Yr15 as on agarose gel. **(B)** KlusterCaller output view of segregation of Co-dominant *Ta5DS-2754099_kasp23* markers in F_3_ plants derived from cross HGW550 + Yr15 × PBW550 + *Lr57-Yr40*. FAM tailed *Lr57-Yr40* (blue color) allele on *X*-axis, HEX tailed PBW550 alleles (red color) on *Y*-axis, heterozygous individuals on mid-axis (green color). Black color represents non-template control and pink color represents unamplified or unclustered samples.

Supplementary Table 1 Raw data of thousand grain weight in three-year replicated trials as recorded on BC_2_F_3–5_ progenies of cross PBW343 X Rye Sel 111 and BC_2_F_3–5_ progenies of cross PBW550 × HGW343 as Env1, Env2 and Env3. The Env 4 Represents the adjusted means of the three years.
