# Supplementary material for: Pyramiding of High Grain Weight With Stripe Rust and Leaf Rust Resistance in Elite Indian Wheat Cultivar Using a Combination of Marker Assisted and Phenotypic Selection
Source: Front Genet. 2020 Dec 22;11:593426. doi: 10.3389/fgene.2020.593426 (PMC7783403; doi:10.3389/fgene.2020.593426)
Supplement: Supplementary Figure 1 — (A) PCR amplification profile of marker Xuhw302 amplifying Yr15 gene in F3 plants derived from cross HGW550 × PBW550 + Yr15 as on agarose gel. (B) KlusterCaller output view of segregation of Co-dominant Ta5DS-2754099_kasp23 markers in F3 plants derived from cross HGW550 + Yr15 × PBW550 + Lr57-Yr40. FAM tailed Lr57-Yr40 (blue color) allele on X-axis, HEX tailed PBW550 alleles (red color) on Y-axis, heterozygous individuals on mid-axis (green color). Black color represents non-template control and pink color represents unamplified or unclustered samples. [file Data_Sheet_1.docx]

**Supplementary Information**

**
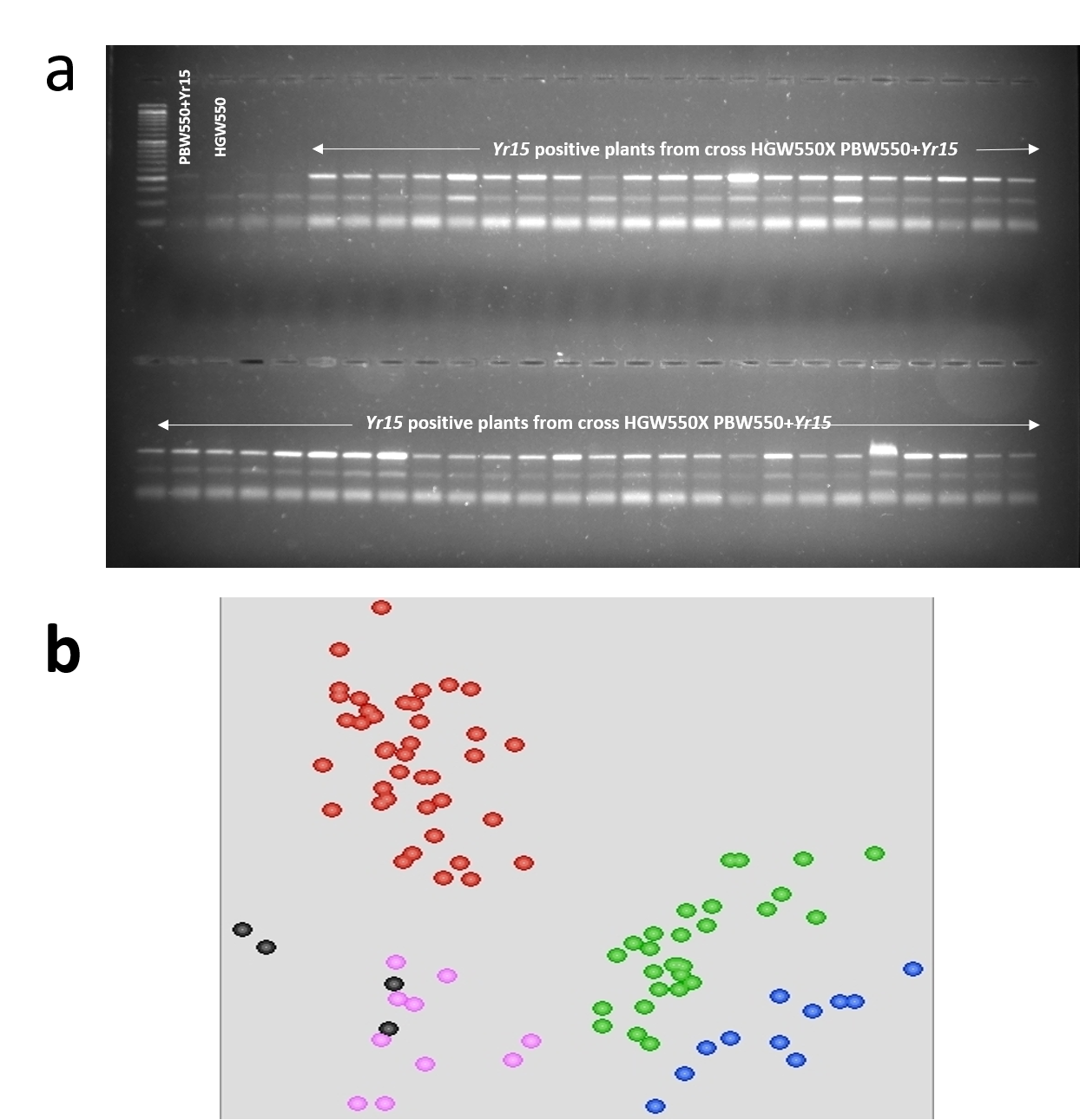
**

**Supplementary Figure S1:** (a) PCR amplification profile of marker *Xuhw302* amplifying Yr15 gene in F_3_ plants derived from cross HGW550 X PBW550+Yr15 as on agarose gel.

(b). KlusterCaller output view of segregation of Co-dominant Ta5DS-2754099_kasp23 markers in F_3_ plants derived from cross HGW550+Yr15 X PBW550+Lr57-Yr40. FAM tailed Lr57-Yr40 (blue colour) allele on X-axis, HEX tailed PBW550 alleles (red colour) on Y-axis, heterozygous individuals on mid-axis (green colour). Black colour represents non-template control and pink colour represents unamplified or unclustered samples.

**Supplementary Table S1:** Raw data of thousand grain weight in three-year replicated trials as recorded on BC_2_F_3-5_ progenies of cross PBW343 X Rye Sel 111 and BC_2_F_3-5_ progenies of cross PBW550 X HGW343 as Env1, Env2 and Env3. The Env 4 Represents the adjusted means of the three years

| **Taxa** | **HGW343** | | | | **HGW550** | | | |
| --- | --- | --- | --- | --- | --- | --- | --- | --- |
|  | **Env1** | **Env2** | **Env3** | **Env4** | **Env1** | **Env2** | **Env3** | **Env4** |
| GW-2 | 48.4 | 46.9 | 48.4 | 45.36336 | 39.3 | 44.97 | 50.64 | 44.91622 |
| GW-3 | 44.7 | 46.3 | 43.7 | 44.34789 | 39.8 | 48.54 | 57.27 | 47.64422 |
| GW-4 | 44.6 | 39.4 | 38.5 | 40.87351 | 35.1 | 44.49 | 53.87 | 44.54653 |
| GW-5 | 45.4 | 43.9 | 48.3 | 45.54399 | 36.3 | 45.84 | 55.37 | 45.5791 |
| GW-6 | 38.3 | 37.1 | 36.3 | 37.79784 | 40.7 | 49.13 | 57.56 | 48.09804 |
| GW-7 | 46.7 | 48.3 | 48.3 | 45.07857 | 42.7 | 48.4 | 54.09 | 47.53714 |
| GW-8 | 39.1 | 37.2 | 36.9 | 38.22501 | 40.9 | 51.4 | 61.89 | 49.83172 |
| GW-9 | 41.1 | 39.7 | 37.9 | 39.79133 | 37.7 | 45.09 | 52.47 | 45.00545 |
| GW-10 | 48.1 | 44.3 | 47.5 | 45.11356 | 35.3 | 43.67 | 52.03 | 43.91935 |
| GW-11 | 39.1 | 48.3 | 43.8 | 43.35114 | 38 | 44.81 | 51.63 | 44.79639 |
| GW-12 | 42.7 | 37.3 | 38.2 | 39.64894 | 40.8 | 47.08 | 53.35 | 46.52752 |
| GW-13 | 38.0 | 35.1 | 35.2 | 36.82957 | 39.6 | 45.36 | 51.11 | 45.21196 |
| GW-14 | 43.8 | 41.2 | 39.0 | 41.30069 | 39.7 | 42.57 | 45.43 | 43.078 |
| GW-16 | 41.3 | 41.0 | 43.9 | 41.92722 | 41.1 | 46.72 | 52.33 | 46.25217 |
| GW-17 | 47.3 | 44.6 | 42.2 | 44.17702 | 40.2 | 45.16 | 50.11 | 45.05899 |
| GW-18 | 43.8 | 39.8 | 41.2 | 41.52852 | 39.5 | 43.97 | 48.44 | 44.15136 |
| GW-19 | 42.3 | 47.5 | 42.5 | 43.66441 | 30.5 | 41.2 | 51.9 | 42.03269 |
| GW-20 | 39.0 | 41.5 | 37.8 | 39.67742 | 36.1 | 43.83 | 51.56 | 44.04428 |
| GW-21 | 44.1 | 39.0 | 37.2 | 40.24699 | 42.4 | 48.35 | 54.3 | 47.50144 |
| GW-22 | 45.9 | 37.1 | 42.5 | 41.72787 | 37.4 | 46.38 | 55.35 | 45.99212 |
| GW-23 | 35.7 | 39.7 | 39.7 | 38.7661 | 37.9 | 47.04 | 56.17 | 46.49693 |
| GW-24 | 43.0 | 39.3 | 37.2 | 40.01916 | 32.4 | 41.4 | 50.4 | 42.18567 |
| GW-25 | 43.0 | 42.7 | 37.3 | 41.01591 | 36.8 | 42.31 | 47.81 | 42.87914 |
| GW-26 | 41.6 | 38.2 | 39.6 | 39.99068 | 33.8 | 42.4 | 50.99 | 42.94798 |
| GW-27 | 43.6 | 47.9 | 42.9 | 44.26245 | 33.4 | 41.35 | 49.29 | 42.14487 |
| GW-29 | 41.7 | 43.7 | 39.7 | 41.61395 | 34.2 | 42.56 | 50.91 | 43.07035 |
| GW-30 | 42.8 | 40.3 | 39.8 | 40.98743 | 35.6 | 42.21 | 48.81 | 42.80265 |
| GW-31 | 40.1 | 42.6 | 39.3 | 40.73112 | 34 | 42.41 | 50.81 | 42.95563 |
| GW-32 | 38.1 | 37.9 | 37.6 | 38.33893 | 34.3 | 44.03 | 53.75 | 44.1947 |
| GW-33 | 41.0 | 37.5 | 37.5 | 39.02241 | 35 | 43.12 | 51.23 | 43.49868 |
| GW-34 | 37.9 | 43.6 | 38.6 | 40.19003 | 36.7 | 47.39 | 58.07 | 46.76463 |
| GW-35 | 38.4 | 39.2 | 41.2 | 39.81981 | 36.3 | 46.58 | 56.86 | 46.14764 |
| GW-37 | 41.2 | 35.3 | 38.3 | 38.68067 | 33.6 | 43.62 | 53.64 | 43.88366 |
| GW-38 | 42.7 | 37.6 | 41.6 | 40.70264 | 37.9 | 45.82 | 53.74 | 45.56635 |
| GW-39 | 39.2 | 39.2 | 35.8 | 38.5098 | 39 | 45.19 | 51.37 | 45.08194 |
| GW-40 | 35.7 | 36.2 | 39.8 | 37.79784 | 40.8 | 46.18 | 51.57 | 45.84425 |
| GW-41 | 37.1 | 40.1 | 45.1 | 40.81656 | 37.5 | 43.91 | 50.31 | 44.10292 |
| GW-42 | 36.9 | 35.9 | 34.9 | 36.6587 | 38.9 | 46.4 | 53.89 | 46.00742 |
| GW-43 | 33.8 | 38.5 | 41.2 | 38.31045 | 41.3 | 44.23 | 47.16 | 44.35022 |
| GW-44 | 36.9 | 35.9 | 34.9 | 36.6587 | 37.7 | 44.62 | 51.54 | 44.64852 |
| GW-46 | 43.0 | 37.2 | 38.0 | 39.64894 | 33.7 | 42.36 | 51.02 | 42.91993 |
| GW-47 | 37.7 | 37.7 | 38.5 | 38.42436 | 34.5 | 43.1 | 51.7 | 43.48593 |
| GW-48 | 45.8 | 36.8 | 41.3 | 41.27221 | 34.3 | 37.62 | 40.94 | 39.29449 |
| GW-49 | 41.4 | 36.7 | 36.4 | 38.59523 | 36.3 | 40.11 | 43.92 | 41.199 |
| GW-50 | 38.6 | 41.2 | 42.2 | 40.73112 | 35.6 | 43.18 | 50.76 | 43.54712 |
| GW-51 | 43.9 | 41.2 | 38.4 | 41.1583 | 38.5 | 44.32 | 50.14 | 44.41906 |
| GW-52 | 40.9 | 40.0 | 38.5 | 39.99068 | 35.3 | 42.49 | 49.67 | 43.01681 |
| GW-54 | 34.6 | 42.3 | 39.6 | 39.1648 | 35.2 | 40.08 | 44.95 | 41.1735 |
| GW-55 | 39.8 | 35.9 | 35.6 | 37.68392 | 34.4 | 43.99 | 53.58 | 44.16665 |
| GW-56 | 36.1 | 42.6 | 39.1 | 39.53502 | 37.9 | 47.58 | 57.25 | 46.90995 |
| GW-57 | 38.1 | 40.3 | 35.3 | 38.36741 | 35.2 | 44.25 | 53.3 | 44.36552 |
| GW-58 | 37.9 | 38.9 | 39.9 | 39.22176 | 36.2 | 46.82 | 57.44 | 46.33121 |
| GW-59 | 40.7 | 43.0 | 38.0 | 40.64569 | 40.4 | 45.97 | 51.54 | 45.68108 |
| GW-60 | 37.2 | 39.9 | 34.9 | 37.88327 | 37.7 | 45.56 | 53.42 | 45.36748 |
| GW-61 | 45.7 | 45.0 | 42.6 | 43.94919 | 34.8 | 43.05 | 51.29 | 43.44514 |
| GW-62 | 40.5 | 37.6 | 37.6 | 38.93698 | 34.6 | 43.77 | 52.93 | 43.99584 |
| GW-63 | 39.0 | 37.4 | 35.8 | 37.94023 | 40.4 | 48.91 | 57.41 | 47.92722 |
| GW-64 | 48.2 | 48.3 | 47.6 | 45.48052 | 32.2 | 42.87 | 53.54 | 43.31001 |
| GW-66 | 48.2 | 48.5 | 43.5 | 45.71161 | 37.7 | 44.05 | 50.39 | 44.21 |
| GW-67 | 43.7 | 42.5 | 37.5 | 41.21526 | 34.8 | 44.7 | 54.59 | 44.70716 |
| GW-68 | 40.8 | 37.3 | 36.5 | 38.62371 | 34.6 | 44.29 | 53.97 | 44.39356 |
| GW-69 | 45.0 | 41.3 | 41.0 | 42.24048 | 40 | 43.58 | 47.16 | 43.85306 |
| GW-70 | 43.1 | 45.2 | 45.9 | 44.2055 | 35.6 | 46.68 | 57.76 | 46.22413 |
| GW-71 | 41.1 | 42.3 | 45.8 | 42.78157 | 36.1 | 45.7 | 55.3 | 45.47456 |
| GW-73 | 39.1 | 40.7 | 42.0 | 40.67416 | 39.8 | 45.57 | 51.34 | 45.37513 |
| GW-74 | 45.5 | 47.9 | 42.7 | 44.74659 | 38.8 | 47.65 | 56.5 | 46.96604 |
| GW-75 | 45.5 | 47.7 | 42.7 | 44.68963 | 36 | 43.7 | 51.39 | 43.94229 |
| GW-76 | 46.0 | 43.9 | 42.9 | 43.8068 | 34.6 | 43.74 | 52.87 | 43.97289 |
| GW-77 | 37.0 | 37.5 | 39.5 | 38.45284 | 37.8 | 44.89 | 51.97 | 44.85248 |
| GW-78 | 41.3 | 38.5 | 39.3 | 39.90524 | 31.9 | 42.94 | 53.97 | 43.361 |
| GW-80 | 45.1 | 39.5 | 44.4 | 42.72462 | 35.8 | 45.39 | 54.98 | 45.23746 |
| GW-81 | 46.4 | 39.7 | 41.7 | 42.38287 | 38.6 | 46.33 | 54.06 | 45.95643 |
| GW-82 | 46.1 | 42.9 | 39.1 | 42.46831 | 35.2 | 44.45 | 53.7 | 44.51849 |
| GW-83 | 44.2 | 40.3 | 39.2 | 41.21526 | 35.3 | 46.18 | 57.05 | 45.83915 |
